# STAT6 expression in T cells, alveolar macrophages and bronchial biopsies of normal and asthmatic subjects

**DOI:** 10.1186/1476-9255-9-5

**Published:** 2012-03-09

**Authors:** Katsuyuki Tomita, Gaetano Caramori, Kazuhiro Ito, Hiroyuki Sano, Sam Lim, Timothy Oates, Borja Cosio, K Fan Chung, Yuji Tohda, Peter J Barnes, Ian M Adcock

**Affiliations:** 1 Department of Respiratory Medicine and Allergology, Kinki University School of Medicine, Osaka, Japan; 2 Section of Respiratory Diseases, Department of Clinical and Experimental Medicine, Università di Ferrara, Ferrara, Italy; 3 Airway Disease Section, National Heart and Lung Institute, Imperial College of London, London, UK

**Keywords:** Airway epithelial cells, Alveolar macrophages, Asthma, STAT6, T-cells, Th2 cells

## Abstract

**Background:**

Asthma is characterised by increased numbers of Th2-like cells in the airways and IgE secretion. Generation of Th2 cells requires interleukin (IL)-4 and IL-13 acting through their specific receptors and activating the transcription factor, signal transducer and activator of transcription 6 (STAT6). STAT6 knockout mice fail to produce IgE, airway hyperresponsiveness and bronchoalveolar lavage eosinophilia after allergen sensitisation, suggesting a critical role for STAT6 in allergic responses.

**Methods:**

We have investigated the expression of STAT6 in peripheral blood T-lymphocytes, alveolar macrophages and bronchial biopsies from 17 normal subjects and 18 mild-moderate steroid-naïve stable asthmatic patients.

**Results:**

STAT6 expression was variable and was detected in T-lymphocytes, macrophages and bronchial epithelial cells from all subjects with no difference between normal and stable asthmatic subjects.

**Conclusions:**

STAT6 expression in different cells suggests that it may be important in regulating the expression of not only Th2-like cytokines in T cells of man, but may also regulate STAT-inducible genes in alveolar macrophages and airway epithelial cells.

## Introduction

Asthma is characterised by chronic airway inflammation, with infiltration of T-lymphocytes, mast cells, eosinophils and monocytes/macrophages. This is associated with the increased expression of several inflammatory proteins, including cytokines, enzymes, receptors and adhesion molecules [1]. The molecular pathways involved in the induction of chronic cytokine expression and recruitment to the airways and activation of inflammatory cells in asthma are not well understood. However, there is increasing recognition that these processes involve increased transcription of inflammatory genes, and that this is regulated by transcription factors [1]. Several transcription factors are involved in asthmatic inflammation including nuclear factor-κB (NF-κB) [2,3] and activator protein-1 (AP-1) [4].

CD4+ T helper (Th) cells can be divided into four major subsets termed Th1, Th2, Th17 and Th0 based on the pattern of cytokines they produce. More recently, another two subsets of effector CD4+ Th cells, named Th9 and Th22 cells, have been described, even if their pathophysiological meaning is still unclear [5,6]. Th1 cells produce predominantly interferon gamma (IFNγ) and predominantly promote cell-mediated immune responses, whereas Th2 cells, which produce mainly IL-4, IL-5 and IL-13, provide help for some B cell responses. IL-4 and IL-13 in particular are the major inducers of B cell switching to IgE production, and therefore play a crucial role in allergic reactions involving IgE and mast cells including bronchial asthma [7]. Th0 cells produce both Th1 and Th2 type cytokines and are precursors to Th1 and Th2 cells [5,6]. Th17 cells release IL-17A, IL-17F and have been implicated in neutrophils recruitment and more severe disease [8]. Of note, a substantial proportion of human Th17 cells produce IFNγ in addition to IL-17A, and these cells were named Th17/Th1 [5].

Recent data suggest the involvement of several transcription factors in the molecular mechanisms by which Th1 and Th2 cells differentially express Th1 and Th2 cytokines genes. For example, differentiation of Th2 cells requires activation of GATA-3 [9] and signal transducer and activator of transcription 6 (STAT6) [10] in mice. Binding of IL-4 and IL-13 with their receptors activates at least two distinct signal transduction pathways, which regulate transcription of specific STAT6-responsive genes. One pathway is associated with the activation of Janus kinase (JAK) 1, 2 and 3. JAK1-3 kinases can, in turn, phosphorylate STAT6 at tyrosine 641, which subsequently forms biologically active homodimers that move from the cytoplasm to the nucleus and regulate transcription of specific STAT6-responsive genes [11]. Generation of Th2-like cells and IgE secretion by IL-4 and IL-13 are mediated by STAT6 in mice [12]. In fact, STAT6 knockout mice have no response to IL-4 and IL-13, do not develop Th2 cells in response to IL-4, fail to produce IgE, airway hyperresponsiveness and bronchoalveolar lavage eosinophilia following allergen sensitisation [13]. This demonstrates the critical role of the STAT6 pathway in allergic responses in mice [14].

Previous data investigating the localisation of STAT6 in the airways of man has produced divergent results. In two studies STAT6 is present only within infiltrating cells of the nose and bronchial mucosa [15,16], whilst in another study STAT6 is expressed predominantly within the bronchial epithelium of mild asthmatic subjects [17].

In order to confirm the site of STAT6-responsive gene expression and activation we have investigated the expression of STAT6 and phosphorylated (activated) STAT6 proteins in peripheral venous blood T cells, alveolar macrophages and bronchial biopsies of normal and asthmatic subjects using Western blotting and immunolocalisation.

## Materials and methods

### Patients

Eighteen mild-moderate asthmatic patients who fulfil the American Thoracic Society Criteria for asthma [18] and 17 age and sex-matched normal subjects were recruited at the Airway Disease Section of the National Heart and Lung Institute of London. The characteristics of normal and asthmatic subjects are summarised in Table 1. All asthmatic patients demonstrated a > 12% improvement in forced expiratory volume in one second (FEV_1_) following inhalation of 200 μg of salbutamol and airway hyperresponsiveness to methacholine with a provocative concentration of methacholine producing a 20% fall in FEV_1 _(PC_20_) of < 8 mg/ml. All asthmatic patients were atopic as defined by two or more positive skin prick tests to common aeroallergens. All asthmatic patients had stable asthma and had not been receiving inhaled or oral glucocorticoid therapy for at least 1 year, and were using only inhaled -agonists drugs intermittently for relief of symptoms. Current smokers or ex-smokers of more than five pack years and patients with FEV_1 _< 75% predicted were excluded. All normal subjects had normal lung function, negative skin prick tests to common allergens (except one subject), no bronchial hyperresponsiveness to methacholine (PC_20 _> 32 mg/ml) and were non-smokers. The study was approved by the Royal Brompton Hospital Ethics Committee and all subjects gave their informed consent.

**Table 1:** Characteristics of subjects

Normals	AGE	SEX	SKIN-TEST	FEV_1 _(% pred)	FVC (%pred)	PC_20 _(mg/ml)
1	38	M	negative	110	105	> 32
2	32	M	negative	102	108	> 32
3	20	M	negative	98	102	> 32
4	23	M	positive	87	91	> 32
5	20	M	negative	83	85	> 32
6	22	F	negative	112	108	> 32
7	20	M	negative	89	110	> 32
8	33	F	positive	92	97	> 32
9	24	M	negative	98	106	> 32
10	38	M	negative	93	97	> 32
11	22	F	negative	85	92	> 32
12	33	M	negative	106	104	> 32
13	40	M	negative	96	95	N/A
14	36	M	negative	105	101	N/A
15	24	F	negative	98	100	N/A
16	39	F	negative	102	103	N/A
17	23	M	negative	106	107	N/A

**Asthmatics**	AGE	SEX	SKIN-TEST	FEV_1 _(% pred)	FVC (%pred)	PC_20 _(mg/ml)

1	26	F	positive	82	100	N/A
2	24	M	positive	80	84	N/A
3	40	M	positive	82	96	1.2
4	27	F	positive	108	101	4.25
5	33	M	positive	78	97	0.78
6	32	M	positive	88	95	N/A
7	23	F	positive	70	83	0.11
8	37	M	positive	106	100	N/A
9	33	M	positive	96	102	1.14
10	29	M	positive	96	105	N/A
11	28	M	positive	85	98	0.46
12	27	F	positive	77	96	0.36
13	25	F	positive	84	100	2.5
14	30	M	positive	81	90	N/A
15	31	M	positive	82	92	0.25
16	22	M	N/A	95	97	2.32
17	47	M	N/A	100	102	3.48
18	23	M	N/A	95	99	0.54

FEV_1_, forced expiratory volume in one second; FVC, forced vital capacity. PC_20_, provocative concentration of methacholine causing a 20% fall in FEV_1_. N/A = not available

### Peripheral venous T cells separation

Peripheral venous blood (100 ml) was collected (between 08:00 and 09:00 h) into sterile 60 ml syringes containing 5 ml of 100% ACD (dextroglucose and Na_2_H Citrate solution). Erythrocyte sedimentation was increased by mixing the blood 1:1 with a solution containing 20 ml of Elohaes (Baxter, Thetford, UK) and 5 ml of 10% ACD (100% ACD diluted in saline). After sedimentation for 60 minutes at room temperature the plasma fraction was layered on Ficoll Paque (Amersham Pharmacia Biotech, Little Chalfont, UK) and after density gradient centrifugation, mononuclear cells (PBMCs) were isolated. Following isolation of PBMCs, T cells were isolated by negative selection of pan T cells by depletion of B cells, monocytes, NK cells, dendritic cells, early erythroid cells, platelets and basophils using magnetic beads according to the manufacture instructions (Miltenyi Biotec, Bisley, UK), as previously described [19]. In some experiments CCR5+ cells were further isolated from T-cells by immunomagnetic beads and subsequently analyzed for STAT6 expression by immunocytochemistry.

### Fibreoptic bronchoscopy, collection and processing of bronchial biopsies, brushings and bronchoalveolar lavage

Normal subjects and asthmatic patients attended the bronchoscopy suite at 8.30 am after having fasted from midnight and were pre-treated with atropine (0.6 mg IV) and midazolam (5-10 mg IV). Oxygen (3 l/min) was administered via nasal prongs throughout the procedure and oxygen saturation was monitored with a digital oximeter. Using local anaesthesia with lidocaine (4%) to the upper airways and larynx, a fiberoptic bronchoscope (Olympus BF10 Key-Med, Southend, UK) was passed through the nasal passages into the trachea. Further lidocaine (2%) was sprayed into the lower airways, and four bronchial mucosal biopsy specimens were taken from segmental and subsegmental airways of the right lower and upper lobes using size 19 cupped forceps. Bronchial biopsies for immunohistochemistry were immediately placed in optimal cutting temperature (OCT) embedding media, then snap frozen in isopentane, pre-cooled with liquid nitrogen, and stored at -70°C. Bronchial biopsies for Western blot analysis were immediately placed on ice and processed as described later. All biopsies were frozen within 20 minutes of collection. Six-micron sections were placed on poly-L-lysine coated microscope slides, air dried for 30 minutes then wrapped in aluminium foil and stored at -70°C prior to immunostaining.

Brushing of the airway was performed with a 2 mm diameter channel cytology brush (Olympus BC-16 C) inserted via the sampling channel of the bronchoscope and rubbed against the epithelial surface. The brush was retracted and retrieved cells dissociated by vortexing in Ham's F12 nutrient medium. This brushing procedure was repeated 8-10 times. Bronchial epithelial cells smears (10,000 cells/slide) were made using a centrifuge (Shandon Cytospin 3, Southend, UK). The cells were air-dried onto the slides and fixed for 10 min in cold 4% phosphate-buffered paraformaldehyde solution.

Bronchoalveolar lavage (BAL) was performed from the right middle lobe using warmed 0.9% NaCl with 4 successive aliquots of 60 mls. Bronchoalveolar lavage fluid was filtered through sterile gauze and centrifuged at 500 g for 10 minutes. BAL cells were washed twice with sterile HBSS. Cytospins were prepared and stained with May-Grunwald stain for differential cell counts. Cells were > 85% viable as assessed by trypan blue exclusion. Macrophages were purified by adhesion as previously described [19].

### Western blot analysis

Whole cell proteins were extracted from T cells, alveolar macrophages and bronchial biopsies as previously described [19]. In brief, T cells, alveolar macrophages and bronchial biopsies were resuspended with mechanical disruption in 30-100 l of 1 × reporter lysis buffer (Promega, Southampton, UK) with a protease inhibitors cocktail (Roche Molecular Biochemicals, Lewes, UK) and phosphatase inhibitors (sodium fluoride and activated sodium orthovanadate), immediately frozen to -70°C and subjected to 3 freeze-thaw steps. Particulate matter was removed by centrifugation at 12000 × *g *for 10 min at 4°C. Protein concentration was measured in the supernatant by the Bradford method according to the manufacturer's instructions (Bio-Rad Laboratories, Hemel Hempstead, UK). 30 g/lane of whole-cell proteins were subjected to 8% SDS-polyacrylamide gel electrophoresis, and transferred to nitrocellulose filters (Hybond-ECL, Amersham Pharmacia Biotech) by blotting. Filters were blocked for 45 minutes at room temperature in tris-buffered saline (TBS), 0.05% Tween 20, 5% non-fat dry milk. The filters were then incubated with rabbit antihuman STAT6 antibody (Santa Cruz Biotechnology, Santa Cruz, CA, USA) and rabbit anti-phosphorylated human (Tyr641) STAT6 antibody (New England Biolabs, Hitchin, UK) for 1 h at room temperature in TBS, 0.05% Tween 20, 5% non-fat dry milk at dilution of 1:1000. The first antibody is specific for both phosphorylated and non-phosphorylated STAT6, the second antibody detects only pSTAT6 phosphorylated at Tyr641. Neither antibody cross-reacts with other STAT proteins. IL-4 stimulated HeLa cell extracts (New England Biolabs) were used as a positive control. Filters were washed three times in TBS, 0.5% Tween 20 and after incubated for 45 minutes at room temperature with anti-rabbit antibody conjugated to horseradish peroxidase (Dako, Ely, UK) in TBS, 0.05% Tween 20, 5% non-fat dry milk, at dilution of 1:4000. After further three washes in TBS, 0.05% Tween 20 visualisation of the immunocomplexes was performed using the ECL as recommended by the manufacturer (Amersham Pharmacia Biotech). As an internal control we reprobed each filter with an anti-human actin antibody (Santa Cruz Biotechnology).

The 43 kDa (actin) or 100 kDa (STAT6 and pSTAT6) bands were quantified using densitometry with Grab-It and GelWorks software (UVP, Cambridge, UK) and expressed as the ratio with the corresponding actin optical density value of the same lane.

### Immunohistochemistry for STAT6 in the bronchial biopsies

Sections were fixed with cold 4% phosphate-buffered paraformaldehyde solution. The biopsies were washed repeatedly with phosphate-buffered saline (PBS). The sections were treated with 0.1% saponin in PBS. Endogenous peroxidase activity was blocked by incubating slides in 1% hydrogen peroxide (H_2_O_2_) and 0.02% sodium azide in PBS for 1 h, followed by washing in PBS. Non-specific labelling was blocked by coating with blocking serum (0.1 M phosphate buffer containing 1% bovine serum albumin and 10% normal swine serum) for 1 h at room temperature. After washing in PBS the sections were incubated overnight at 4°C with a rabbit polyclonal anti-human STAT6 antibody (Santa Cruz Biotechnology) at dilutions of 1:100 of a 200 μg/ml solution.

For the negative control slides, we did not add the respective primary antibody and in some slides normal rabbit non-specific immunoglobulins (Vector Laboratories, Orton Southgate, UK) were used at the same protein concentration as the primary antibody. After overnight incubation and repeated washing steps with PBS, the sections were subsequently incubated with anti-rabbit biotinylated antibody (1:200 dilution; Dako) for 45 minutes at room temperature. After further washing the sections were subsequently incubated with avidin-horseradish peroxidase (1:200 dilution; Dako) for 45 minutes at room temperature. Slides were then incubated with chromogen-fast diaminobenzidine for 5 minutes, after which they were counterstained in hematoxylin and mounted on mounting medium (DPX).

### Quantification

Counts of positive cells were made on all biopsy sections, and were divided according to whether the positive cells were in the airway epithelium or beneath the epithelium to a depth of 175μm. Counts were made only in areas of intact epithelium. For STAT6 proteins, the number of positive cells was expressed as a percentage of nucleated cells in the epithelium and in the subepithelium in at least 4 fields at x400 magnification. For the inflammatory cells, the number of positive cells was expressed as the number per field. For the epithelium, one field was defined as a length of 175μm, and for the subepithelium one field was defined as an area of 175μm^2^. At least four fields were counted for each subject for the epithelium and subepithelium. An experienced observer made all counts unaware of the clinical status or the origin of the sections.

### Immunocytochemical staining for STAT6 in T cells

Cytospins of T cells were fixed with cold 4% phosphate-buffered paraformaldehyde solution. Cytospins were washed repeatedly with PBS. Endogenous peroxidase activity was blocked by incubating slides in 3% hydrogen peroxide (H_2_O_2_) in methanol for 1 h, followed by washing in PBS. Non-specific labelling was blocked by coating with blocking serum (5% normal goat serum) for 20 minutes at room temperature. The cell membranes were permeabilised adding to the blocking serum 0.5% Triton X-100. After washing in PBS the cells were incubated for 1 h with rabbit polyclonal anti-human STAT6 antibody (Santa Cruz Biotechnology) at dilutions of 1:50 of a 200 μg/ml solution.

For the negative control slides, we did not add the respective primary antibody and in some slides normal rabbit non-specific immunoglobulins (http://www.scbt.com) were used at the same protein concentration as the primary antibody. After incubation and repeated washing steps with PBS, the cells were subsequently incubated with anti-rabbit biotinylated antibody (Vector Laboratories) for 30 minutes at room temperature. After further washing the sections were subsequently incubated with ABC reagent (Vector Laboratories) for 30 minutes at room temperature. Slides were then incubated with chromogen-fast diaminobenzidine for 5 minutes after which they were counterstained in haematoxylin and mounted on permanent mounting medium (DPX).

### Statistical analysis

Data are presented as mean ± SD. Differences between normal and asthmatic subjects were assessed with the Mann-Whitney *U*-test, and a value of *p *< 0.05 was taken as significant.

## Results

### T-Lymphocytes

**Figure 1:**
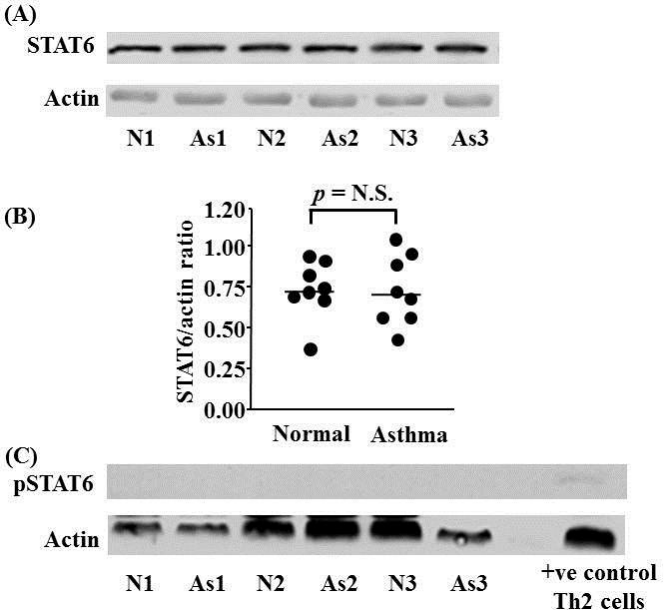
**STAT6 protein expression in peripheral blood T-lymphocytes isolated from asthmatic (As) and normal (N) subjects**. Representative Western blot analyses of experiments from 3 individual subjects in each group are shown (A). Actin expression is used to control for protein loading. Graphical representation of the data in (A) is shown in (B). (C) Failure to detect phospho-STAT6 (pSTAT6) protein expression in peripheral blood T-lymphocytes isolated from As and N subjects. pSTAT6 expression in control Th2 cells confirms the ability of the antibody to detect pSTAT6. Each *circle *represents one subject. *Horizontal bars *represent median values.

**Figure 2:**
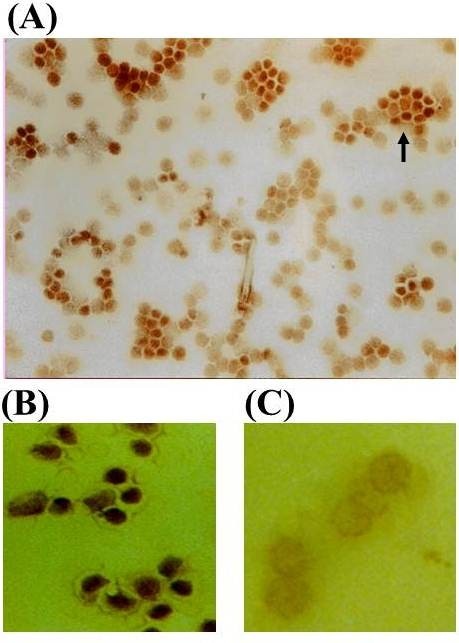
**Representative immunocytochemical staining for STAT6 in T-lymphocytes (A), CCR5- (B) and CCR5+ cells (C)**. STAT6 is localised predominantly to the cytoplasm. However, in a small subset of T cells nuclear localisation of STAT6 can be detected. STAT6 is localised predominantly to the nucleus of CCR5- but not CCR5+ cells.

Western blot analysis of isolated T cells showed no significant difference in the expression of STAT6 in steroid-naive asthmatic patients compared to normal subjects (0.73 ± 0.18 vs 0.72 ± 0.22; not significant) (Figure 1). There was no expression of pSTAT6 in either normal subjects or asthmatic patients (Figure 1C) although a p-STAT6 band was detected in the positive control lane. Immunostaining indicated that STAT6 expression was mainly but not exclusively confined to the cytoplasm. STAT6 was found in the nucleus of some T cells isolated from both normal and mild-moderate steroid-naïve asthmatic subjects (Figure 2A). Using the chemokine receptor CCR5 as a marker for Th1 cells [20] we examined the expression of STAT6 in CCR5+ and CCR5- cells isolated from freshly isolated human T-cells. STAT6 was highly expressed and localised predominantly within the nucleus of CCR5-(Th2-like) cells compared with minimal cytoplasmic expression within CCR5+ (Th1-like) cells (Figure 2B &2C). There was no difference in the number of CCR5+ and CCR5- cells between normal and asthmatic subjects. The pSTAT6 antibody is not able to detect protein by immunocytochemistry or immunohistochemistry (data not shown).

### Alveolar macrophages

**Figure 3:**
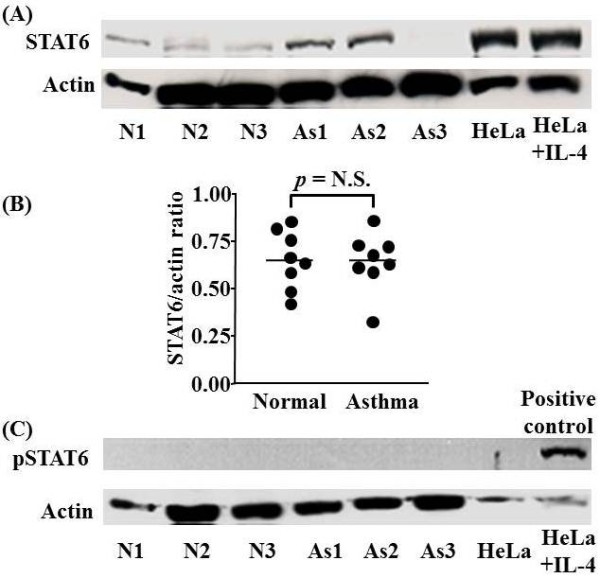
**Representative Western blot analysis of STAT6 and phospho-STAT6 protein expression in alveolar macrophages of normal (N) and asthmatic (As) subjects**. Representative Western blot analyses of experiments from 3 individual subjects in each group are shown (A). Actin expression is used to control for protein loading. Graphical representation of the data in (A) is shown in (B). (C) Failure to detect phospho-STAT6 (pSTAT6) protein expression in alveolar macrophages isolated from As and N subjects. pSTAT6 expression in control IL-4 stimulated HeLa cells confirms the ability of the antibody to detect pSTAT6. Each circle represents one subject. Horizontal bars represent median values.

Using Western blot analysis in alveolar macrophages we observed the presence of STAT6 protein, both in normal subjects and in asthmatic patients. There was no difference in the expression of STAT6 protein between the two groups of subjects (0.65 ± 0.16 vs 0.65 ± 0.16; not significant) (Figure 3). There was no discernable expression of pSTAT6 in either normal subjects or asthmatic patients using Western blotting (Figure 3C) although a p-STAT6 band was detected in the positive control lane.

Representative Western blot analyses of experiments from 3 individual subjects in each group are shown (A). Actin expression is used to control for protein loading. Graphical representation of the data in (A) is shown in B. (C) Failure to detect phospho-STAT6 (pSTAT6) protein expression in alveolar macrophages isolated from As and N subjects. pSTAT6 expression in control IL-4 stimulated HeLa cells confirms the ability of the antibody to detect pSTAT6. Each *circle *represents one subject. *Horizontal bars *represent median values

### Bronchial biopsies

**Figure 4:**
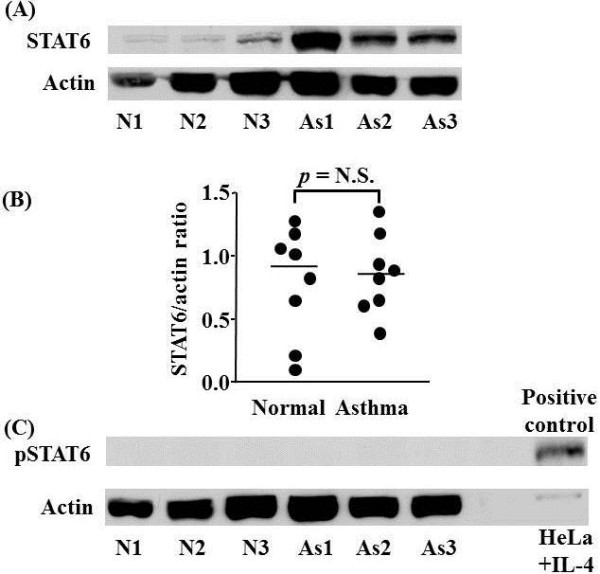
**Western blot analysis of STAT6 and phospho-STAT6 expression in bronchial mucosal biopsies of normal (N) and asthmatic (As) subjects**. Representative Western blot analyses of experiments from 3 individual subjects in each group are shown (A). Actin expression is used to control for protein loading. Graphical representation of the data in (A) is shown in (B). (C) Failure to detect phospho-STAT6 (pSTAT6) protein expression in bronchial biopsies isolated from As and N subjects. pSTAT6 expression in control IL-4 stimulated HeLa cells confirms the ability of the antibody to detect pSTAT6. Each *circle *represents one subject. *Horizontal bars *represent median values.

**Figure 5:**
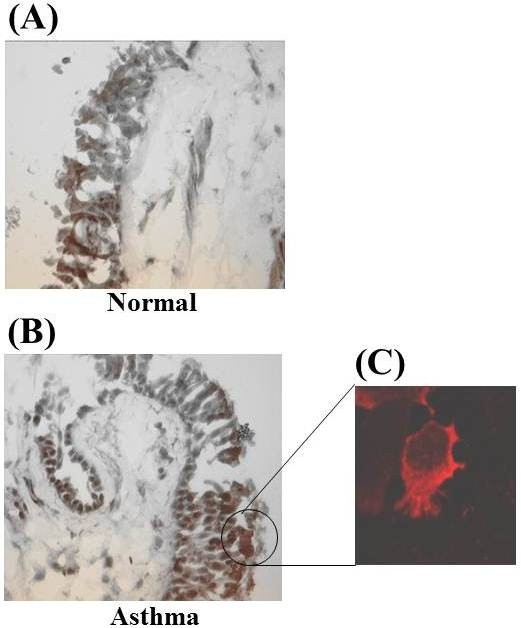
**Immunohistochemical staining for STAT6 protein in bronchial mucosal biopsies of normal (A) and asthmatic (B) subject, and representative immunofluorescence staining for STAT6 in bronchial epithelial cells of an asthmatic subject (C)**.It indicates that STAT6 is localised predominantly to the cytoplasm in bronchial cells of an asthmatic subject.

Immunohistochemical staining (Figure 4) shows that most of the cells staining for the STAT6 protein are bronchial epithelial cells in both normal subjects and asthmatic patients. However, there were some STAT6 positive cells in the lamina propria both in normal and in asthmatic patients (Figure 4). Western blot analysis indicated that there was variable expression of STAT6 but no significant difference in the expression of STAT6 protein (normalised for the actin ratio) in bronchial biopsies of normal subjects compared to asthmatic subjects (0.82 ± 0.5 vs 0.89 ± 0.28; not significant) (Figure 4). Using Western blot analysis we were unable to detect pSTAT6 expression in bronchial biopsies of normal subjects compared with asthmatic patients (Figure 5C) although a p-STAT6 band was detected in the positive control lane.

## Discussion

T cells isolated from peripheral venous blood of normal subjects and steroid-naïve stable asthmatic patients expressed STAT6 protein. However there was no increased expression in T cells from asthmatic patients compared to normal subject or in its activation. This is in accordance with a previous study that found similar levels of STAT6 in PBMCs from normal subjects and patients with extrinsic asthma [21]. This suggests that either there is no increase in activated Th2 cells in the blood in the predominantly mild, stable, asthmatic patients studied here or that STAT6 activation is not important for this process. This further suggests that Th2-like gene regulation in man may be controlled, at least in part by the expression of proteins distinct from those predicted by studies in human cell lines or in the mouse. For example, many animal and human *in vitro *studies suggest that GATA-3 plays an important role in the differentiation of Th2 cells in conjunction with other transcription factors such as nuclear factor of activated T cells (NF-AT)c/B, c-Maf and STAT6 [9,22].

In both normal subjects and asthmatic patients, alveolar macrophages and bronchial epithelial cells also expressed STAT6 proteins. The predominant sites of STAT6 expression in bronchial biopsies are epithelial cells in all subjects. These results are in contrast to two previous studies using immunohistochemistry in which the authors were unable to find any STAT6 immunoreactivity in nasal [15] and bronchial epithelial cells [16] but detected STAT6 localised to the nuclei of some infiltrating cells.

This discrepancy may depend upon differential expression of STAT6 in epithelial cells from upper and lower airways since we found similar results with the two different antibodies by Western blottings. However, we did not use the same STAT6 antibodies (two mouse monoclonals respectively from Transduction Laboratories and Santa Cruz) as Ghaffer and colleagues [15] and Christodoulopoulos and colleagues [16] for immunostaining and their sensitivity may differ. However, this is unlikely to account for the different site of expression seen in bronchial airways. Another study using immunohistochemistry with the same antibody as used here has also shown that the bronchial epithelium is the major site of STAT6 expression [23]. In addition, they also reported no difference in the site of expression between asthmatic and normal control subjects [17].

The presence of STAT6 immunoreactive cells with nuclear localisation of STAT6 in the nasal mucosa of atopic allergic rhinitis may be the result of a selective accumulation of Th2 cells in this disease. This is in accordance with the presence of a minority of T cells with nuclear localisation of STAT6 demonstrated by immunostaining and with the results of a previous study that showed no activation of STAT6 in PBMCs from patients with acute asthma [21].

Human bronchial epithelial cells and alveolar macrophages express IL-4R complex [24], respond to IL-4 and IL-13 and produce cytokines such as GM-CSF and eotaxin which contain STAT6-binding sites in the regulatory sequences of their genes [25-30]. In animal models of allergic asthma, blockade of IL-13 markedly inhibits allergen-induced airway hyperresponsiveness, mucus production and eosinophilia [31]. Furthermore, IL-13 delivery to the airway causes all of these effects [31]. Mice lacking STAT6 gene are protected from all pulmonary effects of IL-13 [31]. Reconstitution of STAT6 only in bronchial epithelial cells is sufficient for IL-13-induced airway hyperresponsiveness and mucus production in the absence of inflammation, fibrosis or other lung pathology [31]. These results demonstrate the importance of direct effects of IL-13 and STAT6 on epithelial cells in causing two central features of bronchial asthma [31].

Interestingly, induction of inducible nitric oxide synthase (iNOS) in both bronchial epithelial cells and alveolar macrophages involves IL-4 and STAT6 [23,32]. These data suggest that STAT6, which is key components of signals via IL-4 and IL-13, may potentially play an important role in modulating inflammatory gene expression in alveolar macrophages and bronchial epithelial cells. However, our result demonstrated the same level of STAT6 in T-lymphocytes, macrophages and bronchial epithelial cells from all subjects with no difference between normal and asthmatic subjects. There are some potential explanations for these results. Firstly, the process of STAT-6 translocation from cytoplasm into nucleus is dependent of other signals, such as Src kinase and phospholipase C activity [33,34]. The existence of cytoplasmic STAT6 might denote that other signals such as oxidative stress, which may induce phospholipase C activity, is required for the translocation of STAT6 into nucleus. Secondly, for IL-4/IL-13-mediated pathway activation, there is also a STAT6-independent process such as a acidic mammalian chitinase [35]. In this paper, STAT6 activation was assessed using immunoblotting. Additional data will be required to assess STAT6 activation by quantative ELISA for identifying transcription factor binding to DNA and chromatin immunoprecipitation assay.

In the last decade many clinical studies have examined the effect of blocking Th2 activity in human asthma. Overall, these studies have proved disappointing in that as a group mild-moderate asthmatics, similar to those studied here, did not respond clinically to drugs targeting IL-4, IL-5 and IL-13 [36-38]. This raises the possibility that the underlying concept of asthma being a Th2-driven hyper-eosinophilic disease which has been developed in animal models is not reflected in human disease except in small subsets of patients with selected asthmatic phenotypes [36,37]. This further highlights the need for more translational research into asthma mechanisms in man if common drivers of disease in human asthma are to be determined.

In summary, T cells from normal subjects and mild-moderate steroid-naïve asthmatic patients in stable phase asthmatics express similar levels of STAT6 protein within each subcellular compartment. Alveolar macrophages and bronchial epithelial cells also express STAT6 proteins with equal expression in normal subjects and asthmatic patients. STAT6 may be important in regulating the expression of Th2-like cytokines in alveolar macrophages and bronchial epithelial cells in addition to T cells in man. Further studies with large number of participants are needed to characterise fully the role of the STAT6 proteins in the regulation of gene expression in these cell types and their potential role in the pathogenesis of asthma, because asthma has various phenotypes. A previous study suggested that treatment with topical glucocorticoids can reduce the number of CD3+/STAT6 immunoreactive cells in nasal mucosa [15]. However at present the ability of glucocorticoids to target STAT6 action in the lower airways is unknown and may determine whether inhibition of STAT6 activity may be a new therapeutic target for anti-asthma drugs.

## Competing interests

The authors declare that they have no competing interests.

## Authors' contributions

KT and GC: Conceived & designed the study & drafted the manuscript. IK, SL and HS: Helped design & participated in the clinical studies & drafting the manuscript. TQ: Participated in the immunohistochemical studies. SL and BC: Helped in clinical samples. KF, YT, PB, and IA: Participated in the study design & coordination and drafting of the manuscript. All authors read and approved the final manuscript.

## References

[B1] Barnes PJ Chung KF Page CP Inflammatory mediators of asthma: an update Pharmacol Rev 1998 50 515 596 9860804

[B2] Hart L Lim S Adcock I Barnes PJ Chung KF Effects of inhaled corticosteroid therapy on expression and DNA-binding activity of nuclear factor kappaB in asthma Am J Respir Crit Care Med 2000 161 224 231 10619824 10.1164/ajrccm.161.1.9809019

[B3] Hart LA Krishnan VL Adcock IM Barnes PJ Chung KF Activation and localization of transcription factor, nuclear factor-kappaB, in asthma Am J Respir Crit Care Med 1998 158 1585 1592 9817712 10.1164/ajrccm.158.5.9706116

[B4] Demoly P Basset SN Chanez P c-fos proto-oncogene expression in bronchial biopsies of asthmatics Am J Respir Cell Mol Biol 1992 7 128 133 1353973 10.1165/ajrcmb/7.2.128

[B5] Annunziato F Galli G Cosmi L Romagnani P Manetti R Maggi E Romagnani S Molecules associated with human Th1 or Th2 cells Eur Cytokine Netw 1998 9 12 16 9831180

[B6] Annunziato F Romagnani S Heterogeneity of human effector CD4+ T cells Arthritis Res Ther 2009 11 257 264 10.1186/ar2843 20053303 PMC3003504

[B7] Bacharier LB Geha RS Molecular mechanisms of IgE regulation J Allergy Clin Immunol 2000 105 Suppl 547 558 10669540 10.1016/s0091-6749(00)90059-9

[B8] Doe C Bafadhel M Siddiqui S Expression of the T helper 17-associated cytokines IL-17A and IL-17 F in asthma and COPD Chest 2010 138 1140 1147 10.1378/chest.09-3058 20538817 PMC2972626

[B9] Ray A Cohn L Th2 cells and GATA-3 in asthma: new insights into the regulation of airway inflammation J Clin Invest 1999 104 985 993 10.1172/JCI8204 10525032 PMC408864

[B10] Kurata H Lee HJ O'Garra A Arai N Ectopic expression of activated Stat6 induces the expression of Th2-specific cytokines and transcription factors in developing Th1 cells Immunity 1999 11 677 688 10.1016/S1074-7613(00)80142-9 10626890

[B11] Schindler C Strehlow I Cytokines and STAT signaling Adv Pharmacol 2000 47 113 174 10582086 10.1016/s1054-3589(08)60111-8

[B12] Daniel C Salvekar A Schindler U A gain-of-function mutation in STAT6 J Biol Chem 2000 275 14255 14259 10.1074/jbc.C000129200 10747856

[B13] Akira S Functional roles of STAT family proteins: lessons from knockout mice Stem Cells 1999 17 138 146 10.1002/stem.170138 10342556

[B14] Tomkinson A Kanehiro A Rabinovitch N Joetham A Cieslewicz G Gelfand EW The failure of STAT6-deficient mice to develop airway eosinophilia and airway hyperresponsiveness is overcome by interleukin-5 Am J Respir Crit Care Med 1999 160 1283 1291 10508820 10.1164/ajrccm.160.4.9809065

[B15] Ghaffar O Christodoulopoulos P Lamkhioued B In vivo expression of signal transducer and activator of transcription factor 6 (STAT6) in nasal mucosa from atopic allergic rhinitis: effect of topical corticosteroids Clin Exp Allergy 2000 30 86 93 10.1046/j.1365-2222.2000.00781.x 10606935

[B16] Christodoulopoulos P Cameron L Nakamura Y TH2 cytokine-associated transcription factors in atopic and nonatopic asthma: evidence for differential signal transducer and activator of transcription 6 expression J Allergy Clin Immunol 2001 107 586 591 10.1067/mai.2001.114883 11295643

[B17] Mullings RE Wilson SJ Puddicombe SM Signal transducer and activator of transcription 6 (STAT-6) expression and function in asthmatic bronchial epithelium J Allergy Clin Immunol 2001 108 832 838 10.1067/mai.2001.119554 11692112

[B18] Standards for the diagnosis and care of patients with chronic obstructive pulmonary disease (COPD) and asthma. This official statement of the American Thoracic Society was adopted by the ATS Board of Directors, November 1986 Am Rev Respir Dis 1987 136 225 244 3605835 10.1164/ajrccm/136.1.225

[B19] Ito K Lim S Caramori G Chung KF Barnes PJ Adcock IM Cigarette smoking reduces histone deacetylase 2 expression, enhances cytokine expression, and inhibits glucocorticoid actions in alveolar macrophages FASEB J 2001 15 1110 1112 11292684

[B20] Di Stefano A Capelli A Lusuardi M Decreased T-lymphocyte infiltration in bronchial biopsies of subjects with severe chronic obstructive pulmonary disease Clin Exp Allergy 2001 31 893 902 10.1046/j.1365-2222.2001.01098.x 11422154

[B21] Miller RL Eppinger TM McConnell D Cunningham-Rundles C Rothman P Analysis of cytokine signaling in patients with extrinsic asthma and hyperimmunoglobulin E J Allergy Clin Immunol 1998 102 503 511 10.1016/S0091-6749(98)70141-1 9768594

[B22] Caramori G Lim S Ito K Expression of GATA family of transcription factors in T-cells, monocytes and bronchial biopsies Eur Respir J 2001 18 466 473 10.1183/09031936.01.00040701 11589343

[B23] Guo FH Uetani K Haque SJ Interferon gamma and interleukin 4 stimulate prolonged expression of inducible nitric oxide synthase in human airway epithelium through synthesis of soluble mediators J Clin Invest 1997 100 829 838 10.1172/JCI119598 9259582 PMC508255

[B24] van der Velden VH Naber BA Wierenga-Wolf AF Interleukin 4 receptors on human bronchial epithelial cells. An in vivo and in vitro analysis of expression and function Cytokine 1998 10 803 813 10.1006/cyto.1998.0365 9811535

[B25] Bonecchi R Facchetti F Dusi S Induction of functional IL-8 receptors by IL-4 and IL-13 in human monocytes J Immunol 2000 164 3862 3869 10725748 10.4049/jimmunol.164.7.3862

[B26] Hart PH Bonder CS Balogh J Dickensheets HL Donnelly RP Finlay-Jones JJ Differential responses of human monocytes and macrophages to IL-4 and IL-13 J Leukoc Biol 1999 66 575 578 10534111

[B27] Matsukura S Stellato C Plitt JR Activation of eotaxin gene transcription by NF-kappa B and STAT6 in human airway epithelial cells J Immunol 1999 163 6876 6883 10586089

[B28] Striz I Mio T Adachi Y Robbins RA Romberger DJ Rennard SI IL-4 and IL-13 stimulate human bronchial epithelial cells to release IL-8 Inflammation 1999 23 545 555 10.1023/A:1020242523697 10565568

[B29] Striz I Mio T Adachi Y Heires P IL-4 induces ICAM-1 expression in human bronchial epithelial cells and potentiates TNF-alpha Am J Physiol 1999 277 L58 L64 10409231 10.1152/ajplung.1999.277.1.L58

[B30] Matsukura S Stellato C Georas SN Interleukin-13 upregulates eotaxin expression in airway epithelial cells by a STAT6-dependent mechanism Am J Respir Cell Mol Biol 2001 24 755 761 11415942 10.1165/ajrcmb.24.6.4351

[B31] Kuperman DA Huang X Koth LL Direct effects of interleukin-13 on epithelial cells cause airway hyperreactivity and mucus overproduction in asthma Nat Med 2002 8 885 889 12091879 10.1038/nm734

[B32] Nemoto Y Otsuka T Niiro H Differential effects of interleukin-4 and interleukin-10 on nitric oxide production by murine macrophages Inflamm Res 1999 48 643 650 10.1007/s000110050516 10669116

[B33] Perez-G M Melo M Keegan AD Zamorano J Aspirin and salicylates inhibit the IL-4- and IL-13-induced activation of STAT6 J Immunol 2002 168 1428 1434 11801685 10.4049/jimmunol.168.3.1428

[B34] Zamorano J Rivas MD Garcia-Trinidad A Qu CK Keegan AD Phosphatidylcholine-specific phospholipase C activity is necessary for the activation of STAT6 J Immunol 2003 171 4203 4209 14530343 10.4049/jimmunol.171.8.4203

[B35] Zhu Z Zheng T Homer RJ Acidic mammalian chitinase in asthmatic Th2 inflammation and IL-13 pathway activation Science 2004 304 1678 1682 10.1126/science.1095336 15192232

[B36] Adcock IM Caramori G Chung KF New targets for drug development in asthma Lancet 2008 372 1073 1087 10.1016/S0140-6736(08)61449-X 18805336

[B37] Caramori G Groneberg D Ito K Casolari P Adcock IM Papi A New drugs targeting Th2 lymphocytes in asthma J Occup Environ Med 2008 3 Suppl 1 6 35 10.1186/1745-6673-3-S1-S6 PMC2259400 18315837

[B38] Nguyen TH Casale TB Immune modulation for treatment of allergic disease Immunol Rev 2011 242 258 271 10.1111/j.1600-065X.2011.01034.x 21682751

